# Clinical Relevance of [^18^F]Florbetaben and [^18^F]FDG PET/CT Imaging on the Management of Patients with Dementia

**DOI:** 10.3390/molecules26051282

**Published:** 2021-02-26

**Authors:** Damiano Librizzi, Nicole Cabanel, Maxim Zavorotnyy, Elisabeth Riehl, Tilo Kircher, Markus Luster, Behrooz Hooshyar Yousefi

**Affiliations:** 1 Department of Nuclear Medicine, Philipps-University of Marburg, 35043 Marburg, Germany; librizzi@staff.uni-marburg.de (D.L.); elisabeth.riehl@posteo.de (E.R.); luster@med.uni-marburg.de (M.L.); 2 Department of Psychiatry and Psychotherapy, Philipps-University of Marburg, 35039 Marburg, Germany; nicole.cabanel@med.uni-marburg.de (N.C.); Maxim.Zavorotnyy@pdag.ch (M.Z.); kircher2@staff.uni-marburg.de (T.K.); 3 Marburg Center for Mind, Brain and Behavior—MCMBB, University of Marburg, 35032 Marburg, Germany; 4 Department of Psychiatry and Psychotherapy, Psychiatric Services Aargau, Academic Hospital of the University of Zurich, 5210 Windisch, Switzerland

**Keywords:** dementia, Alzheimer’s Disease, β-amyloid plaques, neurofibrillary tangles, MCI, positron emission tomography (PET), diagnostic imaging, patient management

## Abstract

PET of β-Amyloid plaques (Aβ) using [^18^F]florbetaben ([^18^F]FBB) and [^18^F]fluorodeoxyglucose ([^18^F]FDG) increasingly aid clinicians in early diagnosis of dementia, including Alzheimer’s disease (AD), frontotemporal disease, dementia with Lewy bodies, and vascular dementia. The aim of this retrospective analysis was to evaluate clinical relevance of [^18^F]FBB, [^18^F]FDG PET and complimentary CSF measurements in patients with suspected dementia. In this study, 40 patients with clinically suspected or history of dementia underwent (1) measurement of Aβ peptides, total tau, and p-tau protein levels in the cerebrospinal fluid (CSF) compared with healthy controls (HC); (2) clinical and neuropsychological assessment, which included Consortium to Establish a Registry for Alzheimer’s Disease neuropsychological assessment battery (CERAD-NAB); (3) [^18^F]FBB and [^18^F]FDG PET imaging within an average of 3 weeks. The subjects were within 15 days stratified using PET, CSF measurements as HC, mild cognitive impaired (MCI) and dementia including Alzheimer´s disease. The predictive dementia-related cognitive decline values were supporting the measurements. PET images were evaluated visually and quantitatively using standard uptake value ratios (SUVR). Twenty-one (52.5%) subjects were amyloid-positive (Aβ+), with a median neocortical SUVR of 1.80 for AD versus 1.20 relative to the respective 19 (47.5 %) amyloid-negative (Aβ-) subjects. Moreover, the [^18^F]FDG and [^18^F]FBB confirmed within a sub-group of 10 patients a good complimentary role by correlation between amyloid pathology and brain glucose metabolism in 8 out of 10 subjects. The results suggest the clinical relevance for [^18^F]FBB combined with [^18^F]FDG PET retention and CFS measurements serving the management of our patients with dementia. Therefore, [^18^F]FBB combined with [^18^F]FDG PET is a helpful tool for differential diagnosis, and supports the patients’ management as well as treatment.

## 1. Introduction

Alzheimer’s Disease (AD) is the most common form of dementia and makes up about two-thirds [1,2,3] of all neurodegenerative disorders (NDD), particularly in older people (≥65 years) [4]. Other NDD are vascular dementia, mixed dementia, PD, Lewy body dementia (LBD) or frontotemporal lobar degeneration (FTLD) [2]. Although these diseases present distinctly different clinical and pathological features, many similar mechanisms are involved in them [5].

β-amyloid plaques (Aβ) and tau depositions are considered as pathological hallmarks of AD and implicated in the disease pathogenesis [2,6,7]. According to the amyloid cascade hypothesis, the pathogenesis of AD is as a result of a dysfunction in the production and the secretion of the amyloid precursor protein (APP) over-producing two major Aβ isoforms: Aβ_1–42_ and Aβ_1–40_, which subsequently misfold and aggregate to form β-amyloid plaques [8,9].

Although no causal link between Aβ plaques deposition and dementia has been established, a definitive diagnosis of AD still requires a histological Aβ plaques examination of brain autopsy sample as a pathological hallmark of AD dementia [10,11,12]. Recent studies suggested that cerebrospinal fluid (CSF) biomarkers, amyloid positron emission tomography (PET), and [^18^F]FDG PET may help early diagnosis of AD [13,14].

The measurement of Aβ peptides and total tau protein levels in the CSF according the European Medicines Agency (EMA) is a complementary usable tool in the diagnosis and therapy monitoring of AD [15,16]. It is a less expensive assessment method, nonetheless needs a careful lumbar puncture in order to reduce the risk of associated side effects and discomfort [17,18,19].

PET has been widely used to help identifying either patients who were at risk of developing AD, and also to monitor disease progression or both [20,21,22]. PET is a very sensitive method, which aids to visualize, characterize, and quantify physiological activities at molecular and cellular levels [23,24]. Hence, amyloid PET may show continued build-up of amyloid deposition beyond the CSF plateau [25]. Therefore, it serves as an important diagnostic tool to provide information on the spatial distribution of the AD pathology and brain metabolism.

[^18^F]FBB also known as AV-1, BAY94-9172 or NeuraCeq was selected because no binding to postmortem cortex of subjects with FTLD or with tauopathies and α-synucleinopathies was observed [26,27,28] and it was a suitable tracer for differential diagnosis in human studies [29]. [^18^F]FBB has shown good sensitivity and specificity for the detection of Aβ in preclinical [30] and clinical setup [31]. Therefore, in this work FBB, FDG, and CSF investigations were carried out to distinguish patients with FTLD from AD, and in a variety of neurodegenerative diseases.

## 2. Results

The baseline characteristics of the 40 evaluated patients are summarized in Table 1. The patients with an [^18^F]FBB+ and [^18^F]FBB− result were on average 71 years old. The percentage of female patients was higher in the group with an [^18^F]FBB+ results (52.4% vs. 31.6%), whereas the mean MMSE was slightly lower in patient with [^18^F]FBB+ compared to the group with [^18^F]FBB− result. Out of the 40 patients, CSF assessments were available in 31 patients. In only 2 patients with positive [^18^F]FBB, Aβ_1–42_ in the CSF was reduced. In 2 patients with negative [^18^F]FBB, Aβ_1–42_ in the CSF was also reduced. There were 13 patients with a non-pathological Aβ_1__–__42_ result who were nevertheless [^18^F]FBB positive. Approximately, half of the patients in both groups had an increased, i.e., pathological, p-tau. Only 4 (10.0%) patients were already treated with an antidementive medication prior to the imaging.

The results as obtained from the logistic regression analysis are summarized in Table 2.

For 17 of the 21 investigated patients (81.0%) with a positive [^18^F]FBB result, a therapy with antidementive medication was recommended by the treating psychiatrist. Sixteen (84.2%) patients with a negative [^18^F]FBB status were not treated with antidementive medication. Only in 7 (17.5%) cases, the psychiatrist recommendation was discordant with the [^18^F]FBB result: 3 (15.8%) patients received an antidementive treatment despite the negative [^18^F]FBB status and 4 (19.0%) patients were not treated with an antidementia although the [^18^F]FBB result showed an Aβ increase in the grey matter. The resulting odds ratio of 22.7 (95% CI: 4.96–141.14) was considerably greater than 1.0 (exploratory *p*-value: 0.0002). Thus, the likelihood of being treated with an antidementia was estimated to be nearly 23-fold higher in patients with a positive [^18^F]FBB status compared to patient with a negative [^18^F]FBB result. One should note that the broad confidence interval indicates some uncertainty of the estimate due to the small sample size. Nevertheless, the observed results confirm a trend that the physicians’ treatment recommendation was motivated by the results obtained from the [^18^F]FBB assessment.

In 8 (20%) of 40 patients, therapy with an antidepressant medication was recommended because of an unsure diagnosis (dementia vs. depression) as summarized in Table 3. For 7 of these 8 patients, the [^18^F]FBB status was assessed as negative indicating that the psychiatrist mostly based their decision on the [^18^F]FBB status.

The [^18^F]FDG result was only available in 10 (25%) of the 40 evaluated patients. Therefore, an analysis of the relation between [^18^F]FDG PET and [^18^F]FBB was very limited, so that only frequencies were calculated as depicted in Table 4.

## 3. Discussion

Dementia is a syndrome with specific diagnoses based on causal factors, neuropathological hallmarks, pattern of cognitive impairment, CSF measurements and imaging. EMA guideline suggests for typical AD, the most common form of dementia, the CSF measurements (decreased Aβ_1–42_ together with increased total tau or p-tau) following in-vivo evidence of the pathology (increased tracer retention on Aβ-PET) [32]. FDG-PET has been increasingly used in the clinical practice supporting the diagnosis of AD (at both mild cognitive impairment—MCI—and early dementia stages), FTLD and its variants, as well as VaD and pseudodepressive dementia.

### 3.1. Glucose Metabolism

Recently, the hypometabolism pattern of AD is well defined, and its negative predicted value may help the differential diagnosis when comorbidities like vascular disease or depression are present [33]. In this work, the pattern of hypometabolism in 6 cases of [^18^F]FDG imaging showed indications of a dementia-typical pattern of AD. For 4 of these 6 cases, this pattern was supported by a positive [^18^F]FBB imaging. However, in the remaining 2 patients, the amyloid imaging was negative, suggesting no Alzheimer-type dementia. All 4 patients with negative [^18^F]FDG imaging also showed negative amyloid-imaging. These results suggest an agreement between FDG and FBB in 8 out of 10 subjects. In a similar setup, regional distribution of amyloid deposition (increased tracer retention by [^18^F]FBB PET) and brain hypometabolism (measured by [^18^F]FDG) association was shown using a longitudinal approach over 2-year follow-up of MCI and early AD patients [34]. Hence, it is reported that in early AD, decline in glucose metabolism is quantitatively related to the amyloid deposition. This interrelationships between brain glucose metabolism and amyloid PET is determined in our study.

### 3.2. Amyloid PET Positivity/Negativity

Obviously, the aim of the diagnostic findings is to arrive at a clear binary result in the sense of amyloid positive or amyloid negative imaging; for example, the determination by nuclear medicine clinician that [^18^F]FBB PET scan shows the presence or absence of Aβ plaque in pathologically relevant brain regions. The protocol for the qualitative [^18^F]FBB that governs positivity or negativity must be standardized [35] and conform to the supplier guideline.

The limitations of amyloid PET: (a) the causality of β-amyloid plaques cannot be established solely with amyloid PET yet, and (b) non-AD patients (e.g., frequently by dementia with Lewy bodies) can also show a positive amyloid PET.

CSF biomarkers assays along with multiple PET imaging approach have been investigated in research and clinical setups. This is because [^18^F]FDG PET in a clinical setting can be diagnostically useful when a characteristic pattern of hypometabolism is detected for specific dementia in addition to molecular brain imaging [36]; in particular, by positive amyloid PET to distinguish AD from DLB or by negative amyloid PET for subtypes of FTLD. CFS assessments of amyloid and tau peptides are widely used to screen populations for AD pathology in clinical settings.

The impact of amyloid PET on the management of patients with suspected dementia is an ongoing worldwide research effort in neuroimaging field to estimate and understand the clinical relevance of AD biomarkers in the assessment of cognitive disorders [37]. Indeed, evidences regarding the agreement among biomarkers, their effectiveness in the correct diagnostic definition and the optimal strategy for combining biomarkers, is still scarce. In this study, we have shown the complimentary effect of FDG PET to amyloid-PET, and compared with CSF. The Aβ42/Aβ40 ratio may increase the diagnostic certainty on the clinical thinking and on the decision making when added to the routine diagnostic workup. The relationship between amyloid load and glucose metabolism of patients with AD were in consistence with already published studies [35,38,39].

**Figure 1:**
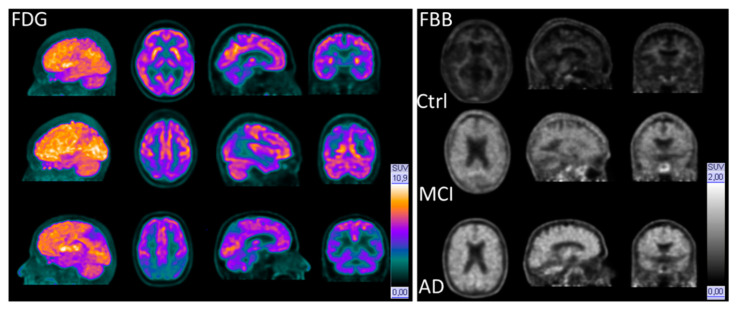
Exemplary 3D, transversal, sagittal, and coronal PET images of healthy control, MCI, and AD (top to down) using [^18^F]FDG (**left**) and [^18^F]FBB (**right**).

CSF results solely cannot help, whereas [^18^F]FBB appears to be a very promising tracer for human amyloid PET imaging. The subjects underwent [^18^F]FDG imaging showed complimentary diagnostic value to the FBB PET (Figure 1). The current CSF and FBB-PET results of dementia patients lead to similar outcome from other groups (Table 5).

## 4. Materials and Methods

### 4.1. Cohort

The human subjects were recruited from the outpatient at the Department of Psychiatry and Psychotherapy of Marburg University. They had been referred for diagnostic evaluation of cognitive impairment and underwent a standardized diagnostic protocol including a comprehensive neuropsychological testing, cerebral MRI or CT, CSF diagnostics, and PET/CT procedure. Examinations were part of their routine check-up in the course of the evaluation of the patients’ suspected neurodegenerative disorders. The retrospective and non-interventional design of this study made patient consent unnecessary. The patients′ characteristics are shown in Table 1.

In this retrospective study, we examined forty-nine patients for a deferential diagnosis of dementia syndrome to exclude or find indications of Alzheimer’s disease. Forty of forty-nine patients were examined during their stay at our Psychiatry and Psychotherapy clinic. We collected data on gender, age, time of examination, MMSI, CSF diagnostics (Aβ_1-42_, tau proteins, p-tau, and Aβ ratio) and compared the working hypothesis before and after amyloid imaging. The local Ethic Committee approved this retrospective study (Ref.: ek_mr_yousefi_ 11_ 12_20).

### 4.2. Neuropsychological Diagnostics

Psychometric workup was based on the Consortium to Establish a Registry for AD neuropsychological assessment battery (CERAD-NAB) [40], which includes the Mini-Mental-State Examination (MMSE) [41]. Further parts of the CERAD-NAB are the evaluations of verbal fluency (animal naming), Boston naming test (15 items), word list (learning, delayed recall, and recognition), constructional praxis, trail making test, and phonematic fluency. For all subtests of the CERAD-NAD, only German language versions were used. The complete CERAD-NAB was available for 47 patients.

### 4.3. CSF Diagnostics

We performed the CSF diagnostics according to the German national S3-guidelines for diagnostics and treatment of dementia (ZITAT: S3-Leitlinie Demenz). For lumbal punction, all study subjects underwent a short in-patient treatment in the Department of Psychiatry and Psychotherapy of Marburg University. The basic CSF diagnostic includes cell count, estimation of total protein, lactate, glucose, and albumin values, as well as the analysis of the cerebral immunoglobulin synthesis, and oligoclonal bands. Additionally, values of total and phosphor-tau, Aβ_1–42_, and Aβ-ratio were measured. The basic CSF diagnostics was performed in the Department of Neurology of Marburg University; the measurements of the neurodegeneration parameters took place in the German National Reference Center at Göttingen University.

Results of CSF diagnostics were available in thirty-one patients. In nine patients, no CSF diagnostics were carried out because the puncture was too risky (systemic anticoagulation) or it was rejected. The clinical information from the Department of Psychiatry was available for the evaluation of the results.

### 4.4. [^18^F]FBB and [^18^F]FDG-PET/CT

All subjects underwent [^18^F]FBB-PET/CT; additionally, [^18^F]FDG-PET/CT was performed in eleven cases. Nine patients came from colleagues in private practice, and the course of further therapy management could not be followed completely and was therefore not considered. In all patients, an organic cause of the dementia syndrome was excluded beforehand (hypothyroidism, vitamin deficiency, etc.).

[^18^F]FDG and [^18^F]FBB were received from Bad Berka Hospital Radiopharmacy and Life Radiopharma f-con GmbH.

PET/CT protocols: The [^18^F]FBB (300 ± 14 MBq) was given as intravenous bolus injection and then the indwelling venous cannula catheter was flushed with 0.9% sterile saline solution. The optimal imaging window for [^18^F]FBB was from 90 to 110 min p.i. using Siemens Software.

The [^18^F]FDG -PET/CT was recorded under standardized conditions with a fasting time of 6 h before the start of the examination. The glucose level of all patients was determined by capillary blood test. The [^18^F]FDG -PET/CT was performed up to a maximum blood sugar value of 150 mg/dL. The patients were screened from visual and acoustic stimuli for 30 min in a darkened room. To perform the cerebral [^18^F]FDG -PET/CT, 200 ± 10 MBq [^18^F]FDG were administered. Data acquisition was performed 60 min post injection (p.i.) using a Siemens Biograph 6 TruePoint PET/CT scanner. For attenuation correction, a low-dose CT with 50 mAs was performed immediately before the acquisition. To avoid motion artefacts, the head was placed in a holder and was fixed. The reconstruction of the PET images was performed iteratively (Gaussian, 3 iterations/21subsets).

The image data were acquired according to the recommendations of national and international guidelines (German Society for Nuclear Medicine and EANM [42,43,44]). After the monitor was adjusted (to the cerebellum), the gray matter of the cortex (temporal, frontal, posterior cingulum/precuneus, and parietal cortex) was examined visually. Each of the brain regions, such as lateral temporal cortex, frontal cortex, posterior cingulate cortex/precuneus, and parietal cortex have been visually assessed and scored according to regional cortical tracer uptake (RCTU)/regional cortical tracer binding (RCTB) scoring and brain b-amyloid plaque load (BAPL)scores using already published procedure [44]. BAPL scores of “1” are classified as “b-amyloid-negative PET scan”, and BAPL scores of “2” and “3” as “b-amyloid-positive PET scan”.

### 4.5. Statistical Analysis

The analysis of the data was done descriptively. Frequencies and percentages were provided for categorical data, mean (SD) were calculated for continuous data. In addition, the interrelationship between brain metabolism, measured by [^18^F]FBB retention, and the clinical treatment of patients with symptoms of dementia was investigated by means of a logistic regression model with the clinical treatment (antidementia yes/no) as dependent variable and [^18^F]FBB result as the only independent variable in the model. The odds ratio and its two-sided 95% confidence interval based on the profile-likelihood method were calculated and the exploratory p-value obtained from the logistic regression analysis was provided. The analysis was completed using R version 3.6.2 [45].

## 5. Conclusions

Several Aβ PET tracers have entered clinical investigation stage; three of them, as aforementioned, have been approved by FDA and EMA and commercially available for aiding nuclear medicine physicians. The brain hypometabolism detected by [^18^F]FDG PET lacks pathological specificity but is very valuable for the detection and staging of disease in case the routinely evaluated MMSE values and measured CSF biomarkers are not conclusive enough. Therefore, using a multi-biomarker approach (e.g., CSF, FDG, and FBB) is recommended to add further evidences and support the stratification of those patients who may have unclear diagnoses.

The use of these diagnostic measures in relation of PET has been explored considering the patient management, and benefits based on resource consumption. A positive [^18^F]FBB PET result that raises confidence in the diagnosis of patients with dementia, is likely to result in earlier and appropriate use of specific medications for symptomatic treatment of dementia especially AD, such as acetylcholinesterase inhibitors and memantine. This diagnostic study showed a greater physician confidence in the diagnosis of or exclusion of AD can result in better medication management. Further assessments of the interrelationships between [^18^F]FBB-PET and [^18^F]FDG-PET using larger cohorts, including various neurodegenerative diseases, are planned.

## Figures and Tables

**Table 1:** Patients’ characteristics.

	[^18^F]FBB+ (N = 21)	[^18^F]FBB− (N = 19)	Total (N = 40)
Age, mean (SD)	71.0 (±9.79)	70.6 (±8.23)	70.8 (±8.97)
**Sex, N (%)**			
Female	11 (52.4%)	6 (31.6%)	17 (42.5%)
Male	10 (47.6%)	13 (68.4%)	23 (57.5%)
MMSE *, mean (SD)	19.9 (±4.34)	22.8 (±5.01)	21.2 (±4.83)
**Aβ_1–42_, N (%)**			
Pathological	2 (9.5%)	2 (10.5%)	4 (10.0%)
Normal	13 (61.9%)	14 (73.7%)	27 (67.5%)
Missing	6 (28.6%)	3 (15.8%)	9 (22.5%)
**Aβ Ratio, N (%)**			
Pathological	9 (42.9%)	11 (57.9%)	20 (50.0%)
Normal	6 (28.6%)	5 (26.3%)	11(27.5%)
Missing	6 (28.6%)	3(15.8%)	9 (22.5%)
**Total tau, N (%)**			
Pathological	12 (57.1%)	5 (26.3%)	17 (42.5%)
Normal	3 (14.3%)	11 (57.9%)	14 (35.0%)
Missing	6 (28.6%)	3 (15.8%)	9 (22.5%)
**p-tau, N (%)**			
Pathological	11 (52.4%)	8 (42.1%)	19 (47.5%)
Normal	4 (19.0)	8 (42.1%)	12 (30.0%)
Missing	6(28.6)	3(15.8%)	9(22.5%)
**Treatment before beta-amyloid imaging**			
Antidementia	4 (19.0)	0 (0.0%)	4 (10.0%)
No Antidementia	17 (81.0)	19 (100.0%)	36 (90.0%)

* For 1 patient ([^18^F]FBB−) MMSE was missing.

**Table 2:** Proportion of patients with Antidementia treatment after [^18^F]FBB result.

	[^18^F]FBB+ N (%)	[^18^F]FBB− N (%)
Antidementia	17 (81.0)	3 (15.8)
No antidementia	4 (19.0)	16 (84.2)
Sum	21	19
Odds ratio *	22.67	
(95% confidence interval) *	(4.96; 141.14)	
*p*-value *	0.0002	

* Based on a logistic regression model including [^18^F]FBB status as the only term in the model. Two-sided 95% confidence interval was calculated applying profile-likelihood method.

**Table 3:** Proportion of patients with antidepressant treatment after [^18^F]FBB result.

	N (%) of [^18^F]FBB+	N (%) of [^18^F]FBB−	Total (%)
Antidepressant *	1 (4.8)	7 (36.8)	8 (20.0)
No antidepressant	20 (95.2)	12 (63.2)	32 (80.0)
Sum	21	19	40

* The antidepressants prescribed are duloxetine 60–120 mg, mirtazapine 15–45 mg, venlafaxine 150 mg, lithium 500 mg, escitalopram 10–20 mg, or citalopram 20 mg.

**Table 4:** [^18^F]FDG PET and [^18^F]FBB result.

	[^18^F]FBB+ (N = 21)		[^18^F]FBB− (N = 19)		Total (N = 40)	
	N	(%)	N	(%)	N	(%)
[^18^F]FDG+	4	(19.0)	2	(10.5)	6	(15.0)
[^18^F]FDG−	0	(0.0)	4	(21.1)	4	(10.0)
No [^18^F]FDG PET performed	17	(81.0)	13	(68.4)	30	(75.0)

**Table 5:** Demographic, CSF, and FBB-PET data of dementia patients.

Age, y, Median (SD)	68.6 (±10.4), Female 40 %
CSF t-tau, median (range) (in pg/mL)	876 (555–2200)
CSF p-tau, median (range) (in pg/mL)	121 (63–210)
CSF Aβ42, median (range) (in pg/mL)	501 (427–571)
Neocortical FBB-PET SUVR * (cerebellar), median (range)	1.80 (1.3–2.5)
FBB-PET SUVR (cerebellar), frontal lobe, median (range)	1.78 (1.3–2.6)
FBB-PET SUVR (cerebellar), parietal lobe, median (range)	1.85 (1.3–2.3)
FBB-PET SUVR (cerebellar), temporal lobe, median (range)	1.72 (1,2–2.2)
FBB-PET SUVR (cerebellar), occipital lobe, median (range)	1.83 (1.2–2.5)

* SUVR, standardized uptake value ratio.

## Data Availability

Patient data is not available for ethical and privacy reasons.

## Sample Availability

Not available.
